# *N*-[4-(*p*-Toluenesulfonamido)phen­ylsulfon­yl]acetamide

**DOI:** 10.1107/S1600536809055706

**Published:** 2010-01-09

**Authors:** Muhammad Ashfaq, Islam Ullah Khan, Muhammad Nadeem Arshad, Hamad Ahmad, Muhammad Nadeem Asghar

**Affiliations:** a Department of Chemistry, University of Gujrat, Gujrat 50700, Pakistan; b Materials Chemistry Laboratory, Department of Chemistry, GC University, Lahore 54000, Pakistan; c Department of Chemistry, Forman Christian College (A Chartered University), Ferozpur Road, Lahore 56400, Pakistan

## Abstract

In the title compound, C_15_H_16_N_2_O_5_S_2_, the dihedral between the two aromatic rings is 81.33 (6)°. In the crystal, pairs of N—H⋯O hydrogen bonds link the mol­ecules into centrosymmetric dimers, which are further connected *via* N—H⋯O hydrogen bonds into a chain running along [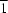
01].

## Related literature

For the synthesis and biological activity of the title compound, see: Deng & Mani (2006 ▶). For a related structure, see: Ashfaq *et al.* (2009 ▶).
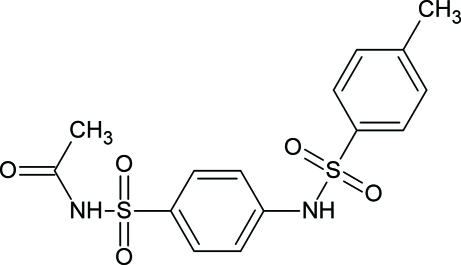


## Experimental

### 

#### Crystal data

C_15_H_16_N_2_O_5_S_2_
*M* *_r_* = 368.42
Monoclinic, 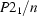

*a* = 9.8077 (4) Å
*b* = 10.0782 (4) Å
*c* = 17.3081 (7) Å
β = 100.290 (2)°
*V* = 1683.28 (12) Å^3^
*Z* = 4
Mo *K*α radiation
μ = 0.34 mm^−1^
*T* = 296 K
0.48 × 0.14 × 0.05 mm

#### Data collection

Bruker Kappa APEXII CCD diffractometer
Absorption correction: multi-scan (*SADABS*; Bruker, 2007 ▶) *T*_min_ = 0.852, *T*_max_ = 0.981
16294 measured reflections
3715 independent reflections
2787 reflections with *I* > 2σ(*I*)
*R*_int_ = 0.032

#### Refinement

*R*[*F* ^2^ > 2σ(*F* ^2^)] = 0.038
*wR*(*F* ^2^) = 0.114
*S* = 1.02
3715 reflections
225 parameters
H atoms treated by a mixture of independent and constrained refinement
Δρ_max_ = 0.34 e Å^−3^
Δρ_min_ = −0.31 e Å^−3^

### 

Data collection: *APEX2* (Bruker, 2007 ▶); cell refinement: *SAINT* (Bruker, 2007 ▶); data reduction: *SAINT*; program(s) used to solve structure: *SHELXS97* (Sheldrick, 2008 ▶); program(s) used to refine structure: *SHELXL97* (Sheldrick, 2008 ▶); molecular graphics: *ORTEP-3 for Windows* (Farrugia, 1997 ▶) and *PLATON* (Spek, 2009 ▶); software used to prepare material for publication: *WinGX* (Farrugia, 1999 ▶) and *PLATON*.

## Supplementary Material

Crystal structure: contains datablocks I, global. DOI: 10.1107/S1600536809055706/bt5152sup1.cif

Structure factors: contains datablocks I. DOI: 10.1107/S1600536809055706/bt5152Isup2.hkl

Additional supplementary materials:  crystallographic information; 3D view; checkCIF report

## Figures and Tables

**Table 1:** Hydrogen-bond geometry (Å, °)

*D*—H⋯*A*	*D*—H	H⋯*A*	*D*⋯*A*	*D*—H⋯*A*
N1—H1*N*⋯O5^i^	0.81 (2)	2.06 (2)	2.848 (2)	162 (2)
N2—H2*N*⋯O1^ii^	0.82 (2)	2.15 (2)	2.950 (2)	167 (2)

Symmetry codes: (i) 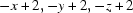; (ii) 
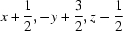
.

**Fig. 1.**
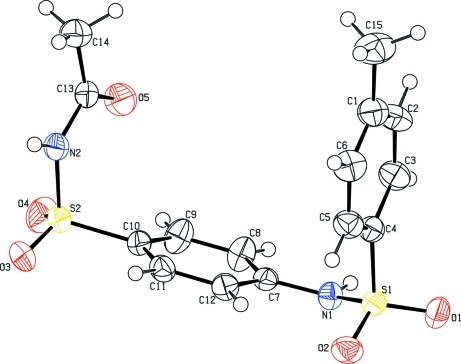
The structure of the title compound with atomic label and displacement ellipsoids drawn at the 50% probability level.

**Fig. 2.**
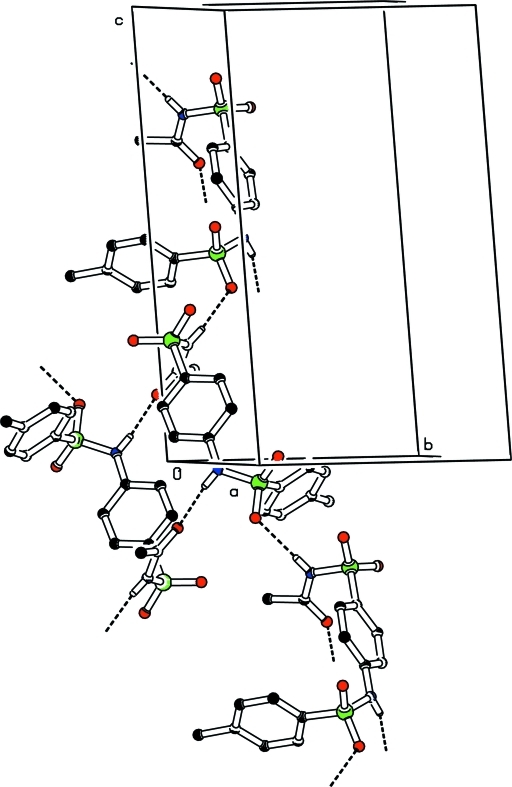
Packing diagram for the title compound with hydrogen bonding drawn as dashed lines. Hydrogen atoms not involve in hydrogen bonding have been omitted.

## supplementary crystallographic information

### Comment

The bond angles and length are in comparison with the previously published crystal structure of N-Acetyl-4-(benzenesulfonamido)benzenesulfonamide (II) (Ashfaq *et al.*, 2009). The dihedral angle between the two aromatic rings is  81.33( 0.06 )° , the acetamido group is oriented at 79.13( 0.11 )° and 14.42 ( 0.26 )° with respect to the central aromatic ring (C7/C8/C9/C10/C11/C12) and toluene ring. The compound may be stabilized by the formation of N–H···O type hydrogen bondings. The acitamido N–H interact with oxygen of C==O moiety and forms a *R*_2_^2^(20) ring. The hydrogen bonding interaction between the sulfonamido N–H and SO_2_ gives rise in the formation of infite long chain along [-1 0 1] (Fig. 2 Table, 1).

### Experimental

The title compound was prepared using a literature method (Deng & Mani, 2006). Sodium sulphacetamide (2 g, 9.3 mmol) was dissolved in distilled water, and then toluene sulfonyl chloride (1.77 g, 9.3 mmol) was added with stirring at room temperature. The pH was maintained at 8-9, strictly using Na2CO3 (1 M). The completion of reaction was observed by the consumption of the suspended toluene sulfonyl chloride. On completion, pH was adjusted to 2-3 using HCl (2 N). The precipitate formed was filtered, washed with distilled water and recrystalyzed from methanol.

### Refinement

The   H-atoms bonded to C were positioned geometrically and refined using a riding model with C–H = 0.93 Å, *U*(H) = 1.2 *U*_eq_(C) for aromatic and C–H = 0.96 Å for CH_3_, *U*(H) = 1.5 *U*_eq_(C) for CH_3_. The N-H H atoms were located in difference map and their coordinates were refined  with *U*(H) = 1.2 *U*_eq_(N) for N atoms.

### Crystal data

**Table:**

C_15_H_16_N_2_O_5_S_2_	*F*(000) = 768
*M**_r_* = 368.42	*D*_x_ = 1.454 Mg m^−^^3^
Monoclinic, *P*2_1_/*n*	Mo *K*α radiation, λ = 0.71073 Å
Hall symbol: -P 2yn	Cell parameters from 4606 reflections
*a* = 9.8077 (4) Å	θ = 2.4–26.8°
*b* = 10.0782 (4) Å	µ = 0.34 mm^−^^1^
*c* = 17.3081 (7) Å	*T* = 296 K
β = 100.290 (2)°	Needle, red
*V* = 1683.28 (12) Å^3^	0.48 × 0.14 × 0.05 mm
*Z* = 4	

### Data collection

**Table:**

Bruker Kappa APEXII CCD diffractometer	3715 independent reflections
Radiation source: fine-focus sealed tube	2787 reflections with *I* > 2σ(*I*)
graphite	*R*_int_ = 0.032
φ and ω scans	θ_max_ = 27.1°, θ_min_ = 2.4°
Absorption correction: multi-scan (*SADABS*; Bruker, 2007)	*h* = −11→12
*T*_min_ = 0.852, *T*_max_ = 0.981	*k* = −12→12
16294 measured reflections	*l* = −22→21

### Refinement

**Table:**

Refinement on *F*^2^	Primary atom site location: structure-invariant direct methods
Least-squares matrix: full	Secondary atom site location: difference Fourier map
*R*[*F*^2^ > 2σ(*F*^2^)] = 0.038	Hydrogen site location: inferred from neighbouring sites
*wR*(*F*^2^) = 0.114	H atoms treated by a mixture of independent and constrained refinement
*S* = 1.02	*w* = 1/[σ^2^(*F*_o_^2^) + (0.0603*P*)^2^ + 0.5119*P*] where *P* = (*F*_o_^2^ + 2*F*_c_^2^)/3
3715 reflections	(Δ/σ)_max_ = 0.001
225 parameters	Δρ_max_ = 0.34 e Å^−^^3^
0 restraints	Δρ_min_ = −0.31 e Å^−^^3^

### Special details

**Table:**

Geometry. All esds (except the esd in the dihedral angle between two l.s. planes) are estimated using the full covariance matrix. The cell esds are taken into account individually in the estimation of esds in distances, angles and torsion angles; correlations between esds in cell parameters are only used when they are defined by crystal symmetry. An approximate (isotropic) treatment of cell esds is used for estimating esds involving l.s. planes.
Refinement. Refinement of F^2^ against ALL reflections. The weighted R-factor wR and goodness of fit S are based on F^2^, conventional R-factors R are based on F, with F set to zero for negative F^2^. The threshold expression of F^2^ > 2sigma(F^2^) is used only for calculating R-factors(gt) etc. and is not relevant to the choice of reflections for refinement. R-factors based on F^2^ are statistically about twice as large as those based on F, and R- factors based on ALL data will be even larger.

### Fractional atomic coordinates and isotropic or equivalent isotropic displacement parameters (Å^2^)

**Table:**

	*x*	*y*	*z*	*U*_iso_*/*U*_eq_	
S1	0.58372 (5)	0.79324 (5)	1.04610 (3)	0.03142 (15)	
S2	0.91252 (5)	0.97819 (6)	0.73350 (3)	0.03660 (16)	
O1	0.55834 (15)	0.82933 (16)	1.12253 (9)	0.0429 (4)	
O2	0.47119 (14)	0.75141 (16)	0.98763 (9)	0.0422 (4)	
O3	0.82154 (16)	0.9285 (2)	0.66630 (9)	0.0553 (5)	
O4	0.96901 (18)	1.10836 (16)	0.73294 (11)	0.0532 (5)	
O5	1.16225 (16)	0.96040 (18)	0.85525 (9)	0.0501 (4)	
N1	0.65425 (17)	0.92526 (18)	1.01581 (10)	0.0315 (4)	
H1N	0.693 (2)	0.971 (2)	1.0518 (14)	0.038*	
N2	1.04204 (18)	0.86957 (19)	0.74560 (10)	0.0341 (4)	
H2N	1.034 (2)	0.811 (2)	0.7122 (14)	0.041*	
C1	0.9121 (2)	0.4715 (2)	1.08120 (15)	0.0427 (5)	
C2	0.9326 (2)	0.5914 (2)	1.12183 (15)	0.0470 (6)	
H2	1.0146	0.6052	1.1572	0.056*	
C3	0.8338 (2)	0.6893 (2)	1.11050 (13)	0.0413 (5)	
H3	0.8485	0.7686	1.1382	0.050*	
C4	0.7115 (2)	0.6690 (2)	1.05720 (12)	0.0316 (4)	
C5	0.6907 (2)	0.5522 (2)	1.01500 (13)	0.0382 (5)	
H5	0.6100	0.5395	0.9784	0.046*	
C6	0.7907 (2)	0.4546 (2)	1.02772 (15)	0.0436 (5)	
H6	0.7761	0.3756	0.9997	0.052*	
C7	0.71121 (19)	0.93381 (19)	0.94715 (11)	0.0278 (4)	
C8	0.8064 (2)	1.0347 (2)	0.94356 (13)	0.0404 (5)	
H8	0.8291	1.0927	0.9856	0.048*	
C9	0.8672 (2)	1.0495 (2)	0.87847 (14)	0.0422 (5)	
H9	0.9305	1.1176	0.8764	0.051*	
C10	0.83393 (19)	0.9629 (2)	0.81628 (12)	0.0306 (4)	
C11	0.7379 (2)	0.8629 (2)	0.81853 (12)	0.0330 (5)	
H11	0.7152	0.8055	0.7761	0.040*	
C12	0.6760 (2)	0.8483 (2)	0.88342 (12)	0.0326 (5)	
H12	0.6110	0.7815	0.8847	0.039*	
C13	1.1547 (2)	0.8753 (2)	0.80521 (12)	0.0354 (5)	
C14	1.2619 (3)	0.7714 (3)	0.80379 (15)	0.0567 (7)	
H14A	1.3395	0.8090	0.7847	0.085*	
H14B	1.2234	0.7003	0.7698	0.085*	
H14C	1.2921	0.7377	0.8559	0.085*	
C15	1.0205 (3)	0.3644 (3)	1.09553 (19)	0.0622 (8)	
H15A	1.1045	0.3959	1.0806	0.093*	
H15B	0.9882	0.2876	1.0649	0.093*	
H15C	1.0381	0.3414	1.1502	0.093*	

### Atomic displacement parameters (Å^2^)

**Table:**

	*U*^11^	*U*^22^	*U*^33^	*U*^12^	*U*^13^	*U*^23^
S1	0.0265 (2)	0.0369 (3)	0.0317 (3)	−0.0007 (2)	0.00741 (19)	0.0050 (2)
S2	0.0365 (3)	0.0453 (3)	0.0291 (3)	0.0072 (2)	0.0089 (2)	0.0104 (2)
O1	0.0458 (9)	0.0506 (10)	0.0363 (9)	0.0046 (7)	0.0186 (7)	0.0054 (7)
O2	0.0288 (7)	0.0490 (9)	0.0467 (9)	−0.0064 (7)	0.0012 (7)	0.0068 (7)
O3	0.0435 (9)	0.0934 (14)	0.0263 (8)	0.0096 (9)	−0.0005 (7)	0.0080 (9)
O4	0.0615 (11)	0.0412 (9)	0.0638 (11)	0.0059 (8)	0.0301 (9)	0.0200 (9)
O5	0.0470 (9)	0.0621 (11)	0.0386 (9)	−0.0020 (8)	0.0001 (7)	−0.0165 (8)
N1	0.0343 (9)	0.0320 (10)	0.0286 (9)	−0.0029 (7)	0.0069 (7)	−0.0014 (7)
N2	0.0366 (9)	0.0396 (11)	0.0259 (9)	0.0044 (8)	0.0051 (7)	−0.0057 (8)
C1	0.0388 (12)	0.0384 (13)	0.0519 (14)	0.0012 (10)	0.0107 (10)	0.0152 (11)
C2	0.0344 (11)	0.0510 (15)	0.0502 (15)	0.0006 (10)	−0.0070 (10)	0.0055 (12)
C3	0.0388 (12)	0.0413 (13)	0.0412 (13)	−0.0034 (10)	0.0004 (9)	−0.0022 (10)
C4	0.0293 (10)	0.0340 (11)	0.0314 (11)	−0.0024 (8)	0.0054 (8)	0.0043 (9)
C5	0.0334 (11)	0.0390 (12)	0.0408 (12)	−0.0047 (9)	0.0024 (9)	0.0011 (10)
C6	0.0458 (13)	0.0312 (12)	0.0541 (15)	−0.0019 (10)	0.0095 (11)	−0.0002 (11)
C7	0.0261 (9)	0.0279 (10)	0.0294 (10)	0.0036 (8)	0.0048 (7)	0.0034 (8)
C8	0.0468 (12)	0.0374 (12)	0.0399 (13)	−0.0130 (10)	0.0157 (10)	−0.0106 (10)
C9	0.0472 (12)	0.0362 (12)	0.0474 (14)	−0.0133 (10)	0.0203 (10)	−0.0059 (10)
C10	0.0299 (10)	0.0337 (11)	0.0290 (10)	0.0040 (8)	0.0077 (8)	0.0049 (9)
C11	0.0343 (10)	0.0363 (12)	0.0270 (10)	−0.0003 (9)	0.0014 (8)	−0.0019 (9)
C12	0.0298 (10)	0.0345 (11)	0.0328 (11)	−0.0069 (8)	0.0035 (8)	−0.0002 (9)
C13	0.0328 (10)	0.0478 (13)	0.0260 (11)	0.0016 (9)	0.0069 (8)	0.0010 (10)
C14	0.0453 (14)	0.080 (2)	0.0434 (14)	0.0217 (13)	0.0050 (11)	−0.0013 (13)
C15	0.0514 (15)	0.0450 (15)	0.092 (2)	0.0109 (12)	0.0188 (14)	0.0226 (15)

### Geometric parameters (Å, °)

**Table:**

S1—O2	1.4215 (15)	C5—C6	1.379 (3)
S1—O1	1.4359 (15)	C5—H5	0.9300
S1—N1	1.6289 (18)	C6—H6	0.9300
S1—C4	1.758 (2)	C7—C8	1.389 (3)
S2—O3	1.4244 (17)	C7—C12	1.394 (3)
S2—O4	1.4247 (18)	C8—C9	1.374 (3)
S2—N2	1.6615 (18)	C8—H8	0.9300
S2—C10	1.751 (2)	C9—C10	1.379 (3)
O5—C13	1.211 (3)	C9—H9	0.9300
N1—C7	1.403 (2)	C10—C11	1.385 (3)
N1—H1N	0.81 (2)	C11—C12	1.377 (3)
N2—C13	1.372 (3)	C11—H11	0.9300
N2—H2N	0.82 (2)	C12—H12	0.9300
C1—C6	1.382 (3)	C13—C14	1.488 (3)
C1—C2	1.394 (4)	C14—H14A	0.9600
C1—C15	1.504 (3)	C14—H14B	0.9600
C2—C3	1.372 (3)	C14—H14C	0.9600
C2—H2	0.9300	C15—H15A	0.9600
C3—C4	1.392 (3)	C15—H15B	0.9600
C3—H3	0.9300	C15—H15C	0.9600
C4—C5	1.381 (3)		
			
O2—S1—O1	119.38 (9)	C1—C6—H6	119.3
O2—S1—N1	109.48 (9)	C8—C7—C12	119.38 (18)
O1—S1—N1	104.07 (9)	C8—C7—N1	117.08 (18)
O2—S1—C4	108.32 (10)	C12—C7—N1	123.53 (18)
O1—S1—C4	108.50 (9)	C9—C8—C7	120.7 (2)
N1—S1—C4	106.35 (9)	C9—C8—H8	119.7
O3—S2—O4	120.38 (11)	C7—C8—H8	119.7
O3—S2—N2	102.99 (10)	C8—C9—C10	119.6 (2)
O4—S2—N2	108.54 (10)	C8—C9—H9	120.2
O3—S2—C10	109.52 (10)	C10—C9—H9	120.2
O4—S2—C10	108.37 (10)	C9—C10—C11	120.42 (19)
N2—S2—C10	106.09 (9)	C9—C10—S2	120.36 (16)
C7—N1—S1	125.38 (15)	C11—C10—S2	119.22 (16)
C7—N1—H1N	114.3 (17)	C12—C11—C10	120.14 (19)
S1—N1—H1N	112.5 (17)	C12—C11—H11	119.9
C13—N2—S2	124.19 (16)	C10—C11—H11	119.9
C13—N2—H2N	121.9 (17)	C11—C12—C7	119.76 (19)
S2—N2—H2N	113.9 (17)	C11—C12—H12	120.1
C6—C1—C2	118.3 (2)	C7—C12—H12	120.1
C6—C1—C15	121.4 (2)	O5—C13—N2	120.4 (2)
C2—C1—C15	120.3 (2)	O5—C13—C14	123.8 (2)
C3—C2—C1	121.1 (2)	N2—C13—C14	115.8 (2)
C3—C2—H2	119.5	C13—C14—H14A	109.5
C1—C2—H2	119.5	C13—C14—H14B	109.5
C2—C3—C4	119.5 (2)	H14A—C14—H14B	109.5
C2—C3—H3	120.2	C13—C14—H14C	109.5
C4—C3—H3	120.2	H14A—C14—H14C	109.5
C5—C4—C3	120.3 (2)	H14B—C14—H14C	109.5
C5—C4—S1	120.96 (16)	C1—C15—H15A	109.5
C3—C4—S1	118.77 (17)	C1—C15—H15B	109.5
C6—C5—C4	119.3 (2)	H15A—C15—H15B	109.5
C6—C5—H5	120.3	C1—C15—H15C	109.5
C4—C5—H5	120.3	H15A—C15—H15C	109.5
C5—C6—C1	121.5 (2)	H15B—C15—H15C	109.5
C5—C6—H6	119.3		
			
O2—S1—N1—C7	−58.48 (18)	C15—C1—C6—C5	179.4 (2)
O1—S1—N1—C7	172.83 (16)	S1—N1—C7—C8	−158.64 (16)
C4—S1—N1—C7	58.34 (18)	S1—N1—C7—C12	22.1 (3)
O3—S2—N2—C13	−178.58 (18)	C12—C7—C8—C9	−0.9 (3)
O4—S2—N2—C13	52.8 (2)	N1—C7—C8—C9	179.8 (2)
C10—S2—N2—C13	−63.5 (2)	C7—C8—C9—C10	−0.3 (4)
C6—C1—C2—C3	1.4 (4)	C8—C9—C10—C11	1.2 (3)
C15—C1—C2—C3	−178.9 (2)	C8—C9—C10—S2	−178.87 (18)
C1—C2—C3—C4	−0.4 (4)	O3—S2—C10—C9	−151.90 (18)
C2—C3—C4—C5	−1.1 (3)	O4—S2—C10—C9	−18.8 (2)
C2—C3—C4—S1	177.79 (18)	N2—S2—C10—C9	97.58 (19)
O2—S1—C4—C5	−2.5 (2)	O3—S2—C10—C11	28.04 (19)
O1—S1—C4—C5	128.50 (18)	O4—S2—C10—C11	161.13 (16)
N1—S1—C4—C5	−120.07 (18)	N2—S2—C10—C11	−82.47 (17)
O2—S1—C4—C3	178.62 (17)	C9—C10—C11—C12	−0.8 (3)
O1—S1—C4—C3	−50.4 (2)	S2—C10—C11—C12	179.30 (16)
N1—S1—C4—C3	61.02 (19)	C10—C11—C12—C7	−0.5 (3)
C3—C4—C5—C6	1.7 (3)	C8—C7—C12—C11	1.3 (3)
S1—C4—C5—C6	−177.20 (17)	N1—C7—C12—C11	−179.41 (18)
C4—C5—C6—C1	−0.7 (3)	S2—N2—C13—O5	2.9 (3)
C2—C1—C6—C5	−0.8 (4)	S2—N2—C13—C14	−177.73 (17)

### Hydrogen-bond geometry (Å, °)

**Table:**

*D*—H···*A*	*D*—H	H···*A*	*D*···*A*	*D*—H···*A*
N1—H1N···O5^i^	0.81 (2)	2.06 (2)	2.848 (2)	162 (2)
N2—H2N···O1^ii^	0.82 (2)	2.15 (2)	2.950 (2)	167 (2)

Symmetry codes: (i) −*x*+2, −*y*+2, −*z*+2; (ii) *x*+1/2, −*y*+3/2, *z*−1/2.
